# Advanced Nanoporous Materials for Micro-Gravimetric Sensing to Trace-Level Bio/Chemical Molecules

**DOI:** 10.3390/s141019023

**Published:** 2014-10-13

**Authors:** Pengcheng Xu, Xinxin Li, Haitao Yu, Tiegang Xu

**Affiliations:** State Key Lab of Transducer Technology, Shanghai Institute of Microsystem and Information Technology, Chinese Academy of Sciences, 865 Changning Road, Shanghai 200050, China; E-Mails: xpc@mail.sim.ac.cn (P.X.); yht@mail.sim.ac.cn (H.Y.); xutiegang@mail.sim.ac.cn (T.X.)

**Keywords:** resonant micro-cantilever, gravimetric sensor, mesoporous silica, graphene, zeolites, functionalization, bio/chemical molecules

## Abstract

Functionalized nanoporous materials have been developed recently as bio/chemical sensing materials. Due to the huge specific surface of the nano-materials for molecular adsorption, high hopes have been placed on gravimetric detection with micro/nano resonant cantilevers for ultra-sensitive sensing of low-concentration bio/chemical substances. In order to enhance selectivity of the gravimetric resonant sensors to the target molecules, it is crucial to modify specific groups onto the pore-surface of the nano-materials. By loading the nanoporous sensing material onto the desired region of the mass-type transducers like resonant cantilevers, the micro-gravimetric bio/chemical sensors can be formed. Recently, such micro-gravimetric bio/chemical sensors have been successfully applied for rapid or on-the-spot detection of various bio/chemical molecules at the trace-concentration level. The applicable nanoporous sensing materials include mesoporous silica, zeolite, nanoporous graphene oxide (GO) and so on. This review article focuses on the recent achievements in design, preparation, functionalization and characterization of advanced nanoporous sensing materials for micro-gravimetric bio/chemical sensing.

## 1. Introduction

Originally, the materials of nanoscale pores, like zeolites and mesoporous materials, were developed as adsorbents and catalysts in the petroleum industry [1–12]. Benefitting from the huge specific surface area, versatile surface functionalization, and the highly ordered and stable nanostructure, the usage of the nanoporous materials has been recently expanded to more attractive fields such as optical, biomedical, electronic, environmental and sensing applications [13–19]. Among those developed materials, nanoporous materials for bio/chemical sensing have drawn much attention [16,20,21]. Aiming at achieving high sensitivity, many researchers have tried to make scientific and technical improvements of nanoporous structures to enhance surface area of sensing materials. For example, Egashira and the co-workers prepared mesoporous tin-dioxide (SnO_2_) to improve the sensitivity of semiconductor-metal-oxide sensors [22,23]. Similarly, one-dimensional metal oxide nanomaterials like nanowires, nanorods and nanotubes were used to construct high-sensitivity materials with nanoporous structure [24–27]. However, many typical nanoporous materials such as mesoporous silica and AAO (anodic aluminum oxide) are electrically nonconductive and unsuitable for direct chemiresistive or semiconductive sensing. Due to this reason, such nanoporous structures have been employed as carrier of the sensing materials. For instance, Li and the co-workers loaded porphyrin onto mesoporous silica to form porphyrin-doped bi-modal porous-silica films. By using this material, they found that the fluorescence quenching efficiency by TNT (trinitrotoluene) increases 57% in 10 s, thereby achieving much higher sensitivity than that of the conventional conjugated-polymer based TNT sensors [28].

**Figure 1.**
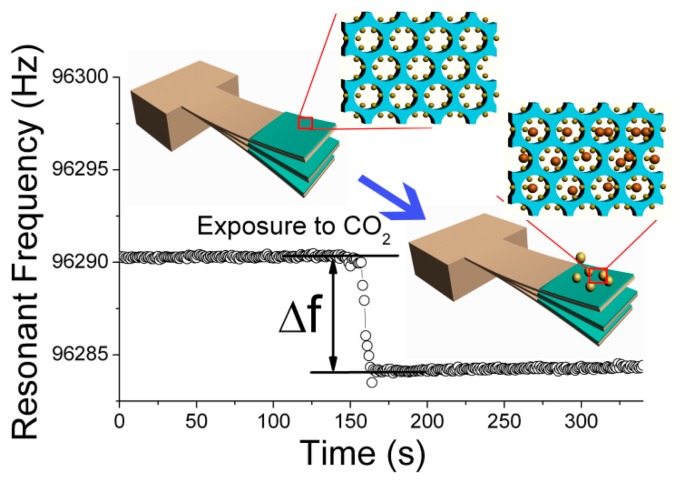
Schematic of micro-gravimetric sensing mechanism of resonant micro-cantilever sensor, at the end of which the nanoporous sensing material is loaded. Reprinted with permission from [35].

Recently, micro/nano cantilever sensors have been utilized to detect bio/chemical molecules in many application fields of bio-medical analysis, environmental monitoring, food safety and so on [29]. Among the various types of cantilevers, the micro/nano-machined gravimetric transducer of resonant micro-cantilever has been considered an ideal micro-platform for sensing-performance research of nanoporous materials [30,31]. The resonant frequency of the cantilever can shift according to the change in either mass, spring constant or surface modification effects [32,33]. By concentrating on the mass change near the cantilever end and with appropriate treatment to the cantilever surface, the frequency shift can be mainly determined by mass change. As is schematically shown in Figure 1, by loading the nanoporous material onto the designated micro-region of the resonant micro-cantilever, the added mass from bio/chemical molecule adsorption onto the nanoporous sensing material can be accurately detected by reading the frequency-shift of the micro resonator. With functionalization at the ultra-high surface area, the nanoporous sensing materials can significantly enhance the micro-gravimetric sensing response and specificity [34,35]. These advanced sensing materials have been broadly used for detection of trace bio/chemical molecules. So far, several kinds of nanoporous materials have been successfully developed for gravimetric detection of trace bio/chemical substances. These sensing materials include functionalized mesoporous silica, zeolite, nanoporous graphene oxide (GO) and so on.

On the other hand, compared with other types of micro-gravimetric transducers like QCM (quartz crystal microbalance), the silicon micro-machined resonant cantilever has merit in its finer mass resolution, more intuitive sensing mechanism, smaller volume, low cost batch fabrication, easy IC compatible integration and so on [36–39]. In addition, the resonant cantilever has been evolved to nanoscale devices for higher sensitivity. As is introduced in recent publications [36,37,40], the limit of detection of nano-cantilever is finer than the attogram (10^−18^ g) in ambient environment. However, the developed nano-cantilevers feature technical drawbacks. Due to surface-to-volume size effect, nano-cantilevers generally have lower Q-factor than micro-cantilevers. Besides, it is difficult to load sensing materials onto the nanometric sensing area of the nano-cantilever. Roukes and his co-researchers reported a method of surface-initiated polymerization to grow thick and uniform poly(methyl methacrylate) sensing-film onto nano-cantilever [40]. Unfortunately, such an *in situ* polymerization method is not suitable for loading other non-polymer materials like zeolites. Currently, micro-sized resonant cantilevers are very suitable for practical applications. In the long term, nano-cantilevers are no doubt the developing trend of the future. Due to the limited scope of this review, we only focus our discussion on the advanced nanoporous sensing-materials for micro-cantilever gravimetric detection of bio/chemical molecules. Moreover, we would like to clearly state that all the addressed nanoporous sensing materials in this review can be used for other types of gravimetric sensing platforms [41–43].

## 2. Integrated Resonant Micro-Cantilevers for Gravimetric Sensing

The design and fabrication of the resonant micro-cantilever have been reported in a very large number of literatures [44–47]. The resonant micro-cantilever can be fabricated by using different materials and designed with various geometries. The developed representative resonant micro-cantilevers are categorized in Table 1.

Herein, aimed at bio/chemical molecule detection, we only introduce two kinds of integrated resonant micro-cantilevers, with one for gaseous molecules detection and another for ion/biological detection in aqueous solutions [48,49]. Both of the cantilevers are integrated with electrothermal exciting and piezoresistive read-out elements. The resonance of the cantilever can be excited by external actuating methods like light exposure [50]. For miniaturization and portable sensing applications, an integrated resistor can be used as a micro-heater to electrothermally excite the resonance. As is introduced in the previous report [48], the cantilever for gas sensing is designed with the dimensions of 200 μm × 100 μm × 3 μm and the resonant frequency of the operation mode (fundamental bending mode) is about 100 kHz. According to the literature [51] where noise of resonant micro-cantilever is discussed, the frequency signal of the cantilever mainly suffers from mechanical-thermal noise, as the cantilever works in ambient atmosphere and its resonant frequency is far below 1 MHz. The other noise sources like temperature fluctuation noise, optical effect and Knudsen effect can be ignored [50,52]. For this electro-thermally excited cantilever, the mechanical-thermal noise brings a very low frequency fluctuation (at level of 10^−3^ Hz) that has been verified by our sensing experiment. As for the cantilever operated in liquid [49], the specially designed micro-cantilever is excited in an in-plane swing mode. Since the resonating structure shears with the surrounding liquid media rather than compressing the media, the damping effect can be effectively depressed and a high Q factor can be achieved in liquid.

### 2.1. Integrated Resonant Micro-Cantilever for Gaseous Molecule Detection

**Figure 2.**
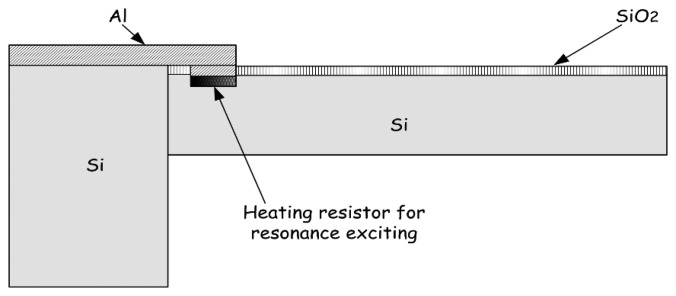
Cross-sectional schematic of a silicon micro-cantilever, in which a resonance exciting heater is integrated. Reprinted with permission from [48].

**Figure 3.**
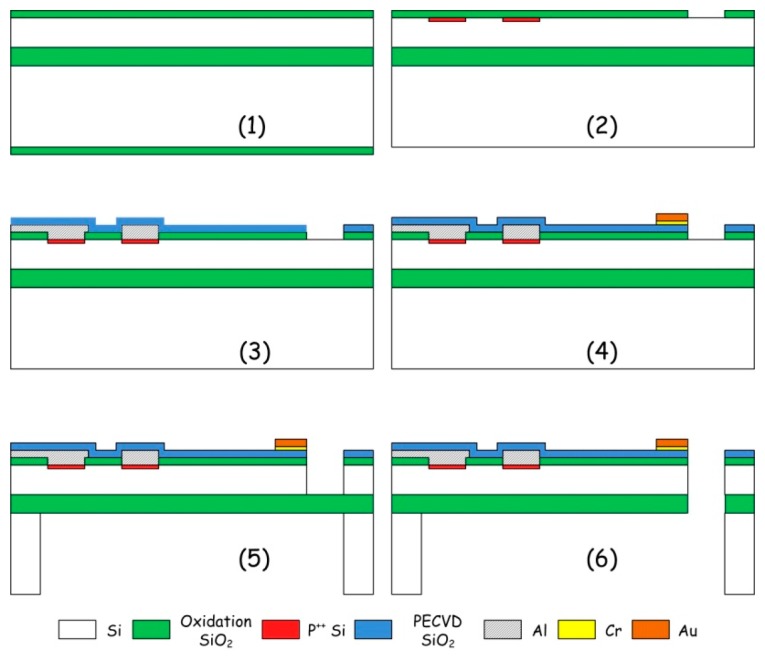
Micromachining fabrication processes of the integrated resonant micro-cantilever. Reprinted with permission from [48].

**Figure 4.**
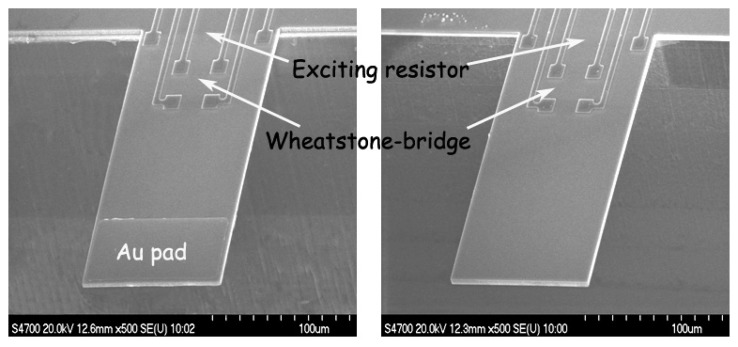
SEM images of the fabricated micro-cantilevers, with the optimally designed resonance exciting resistor and the signal-pickup piezoresistive Wheatstone-bridge integrated near the clamped root of the cantilever. The cantilever dimensions are 200 μm × 100 μm × 3 μm. Reprinted with permission from [48].

For chemical vapor sensing, a resonant micro-cantilever has been optimally designed and fabricated with silicon micromachining techniques, where the resonance exciting micro-heater and the self-sensing piezoresistor are both integrated [48]. Figure 2 shows the cross-sectional schematic of the resonant cantilever. A boron-doped resistor is laid near the root of the cantilever to serve as the micro-heater. Thermal waves were generated by heating the resistor with AC electric current and propagated through micromechanical vibration of the silicon cantilever. The pulsed heating induces thermal expansion and further causes mechanical bending strain of the cantilever. For the silicon cantilever with a SiO_2_ insulating thin film on top, the generated bending strain can be originated from either the difference in thermal expansion coefficients of the Si/SiO_2_ double-layer or the temperature gradient across the cantilever thickness. Figure 3 outlines the fabrication process flow for the integrated resonant cantilever. Figure 4 shows two SEM images of the fabricated micro-cantilevers. Besides the micro-heater, a piezoresistive Wheatstone-bridge is also integrated for frequency signal readout.

### 2.2. Higher-Mode Resonant Micro-Cantilever for Ion/Bio-Molecule Detection

**Figure 5.**
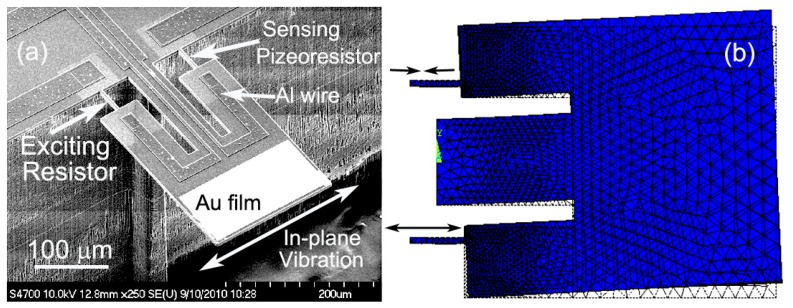
(**a**) SEM image of the proposed and fabricated resonant micro-cantilever sensor that can be operated in in-plane swing resonance mode; (**b**) ANSYS simulation results of the in-plane resonance mode shape. Reprinted with permission from [49].

For real-time bio/chemical detection in liquid environment like aqueous solution, improved resonant cantilever structures is designed that can be operated in higher resonance modes than fundamental bending mode resonance [49]. With lower liquid damping and drag-force effect, the higher-mode resonators can achieve high enough Q-factor for frequency-shift detection in liquid. Compared with bending-mode of conventional cantilever, in-plane resonance mode is advantageous in enduring lower liquid resistive effect and gaining higher Q-factor in liquid. As is shown in the SEM image of Figure 5a, the double tiny-beams structured resonant cantilever is designed and fabricated for sensing in in-plane resonance mode. The in-plane mode resonant sensor consists of a central supporting-beam and a pair of silicon tiny-beams at its both sides, with one for electro-thermal resonance excitation and another for piezoresistive frequency-readout. A thin sensing plate is loaded at the end of the central supporting-beam and linked with the two tiny-beams *via* two overhanging legs of the sensing plate. The resonance exciting resistor and the sensing piezoresistor are embedded in the silicon tiny-beams by boron doping. The distance between the tiny-beam and the central supporting-beam has been optimally designed so that the tiny-beams can only straightly (axially) compress or stretch instead of bending movement. This pure axial vibration of the tiny-beams is achieved by counteracting between the positive lateral movement and the negative rotating movement of the sensing plate when the structure resonates in the in-plane mode. By supplying a pulsed current through the thermal exciting resistor, the pulse-heating induced extensional expansion in longitudinal direction of the tiny-beam will lead the sensing-plate into in-plane swing resonance. At another side, the tiny-beam piezoresistor is forced into pulsed compression and the piezoresistive frequency signal can be electrically read out. The ANSYS-simulated resonance mode shape is illustrated in Figure 5b. When the AC current flows through the exciting tiny-beam to cause the pulsed thermal expansion, the in-plane resonance mode of the sensor is confirmed, while the unwanted out-of-plane resonant modes at lower frequencies can be well depressed.

**Figure 6.**
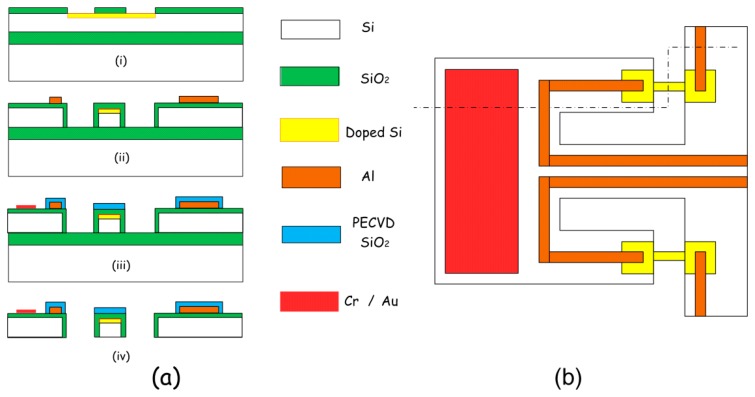
(**a**) Sensor fabrication process; (**b**) Top-view schematic showing the dash-dotted line, along which the cross-section view is cut and shown in (**a**). Reprinted with permission from [49].

Figure 6a depicts the fabrication process of the in-plane mode piezoresistive resonant micro-sensor for gravimetric detection in liquid. Silicon-on-insulator (SOI) wafer with 5 μm-thick n-type (100) device layer is used as the starting material to form the structure that includes the thin sensing plate for low drag force in liquid. The resistivity of the starting n-type device layer is 1∼10 Ω cm. Figure 5b shows the top-view schematic of the micro-sensor. The cross section shown in Figure 6a is cut along the dash-dotted line in Figure 6b. The fabrication process flow for the resonant micro-sensor is detailed as follows. Firstly, a 0.2 μm-thick SiO_2_ layer was thermally grown in dry oxidation and wet oxidation, respectively. After patterned for a 1.4 μm-thick photoresist layer, the 1 μm-thick p-type piezoresistor, resonance exciting resistor and the fixed resistors for constructing a Wheatstone-bridge are all formed by boron ion-implantation with 1 × 10^15^/cm^3^ dose and 75 keV energy, as is shown in step (i). Deep reactive-ion etching (deep RIE) is employed to shape the structure of the sensor from the front side of the wafer. After wet etching to open the contact holes, a 0.5 μm-thick aluminum layer is sputtered and patterned by wet etching for interconnection wires, which is followed by sintering in forming gas at 450 °C for 30 min (see the step of (ii)). A high-quality plasma enhanced chemical-vapor-deposition (PECVD) silicon-dioxide passivation layer is deposited on top of the structure and patterned by reactive-ion etching (RIE) to cover the aluminum lines. The passivation layer can sustain the aluminum against Piranha solution (7/3 of 98% H_2_SO_4_/30% H_2_O_2_) cleaning process that will be done before bio/chemical sensing experiment. The passivation layer also serves as an insulate layer to prevent from electric leakage when the sensor is immersed in conductive solution (see step (iii)). Finally, as is shown in step (iv), the resonant microstructure is released by deep RIE and HF etch from the backside of the wafer to remove the handle layer and the BOX (buried oxide) layer of the SOI wafer.

## 3. Functionalized Mesoporous Silica

Featuring ultra-high specific surface area and a large amount of adsorption sites, mesoporous silica is one of the ideal materials for molecules adsorption [9,15,17,53,54]. Without doubt, the advantage of high surface area can bring large gravimetric sensing response, when it is loaded onto the resonant micro-cantilever. In addition to sensitivity, attention should be paid on another important performance aspect of selectivity. Because functional organic groups have special interaction capability with some target molecules, surface graft of organic group is considered one of the most effective methods to improve selectivity. For example, there is a special interaction between carboxylic group (-COOH) and ammonia molecule [34,55,56]. Based on the surface modification scheme, the mesoporous silica can be pre-functionalized with some specific organic groups to become a specific micro-gravimetric sensing material.

**Figure 7.**
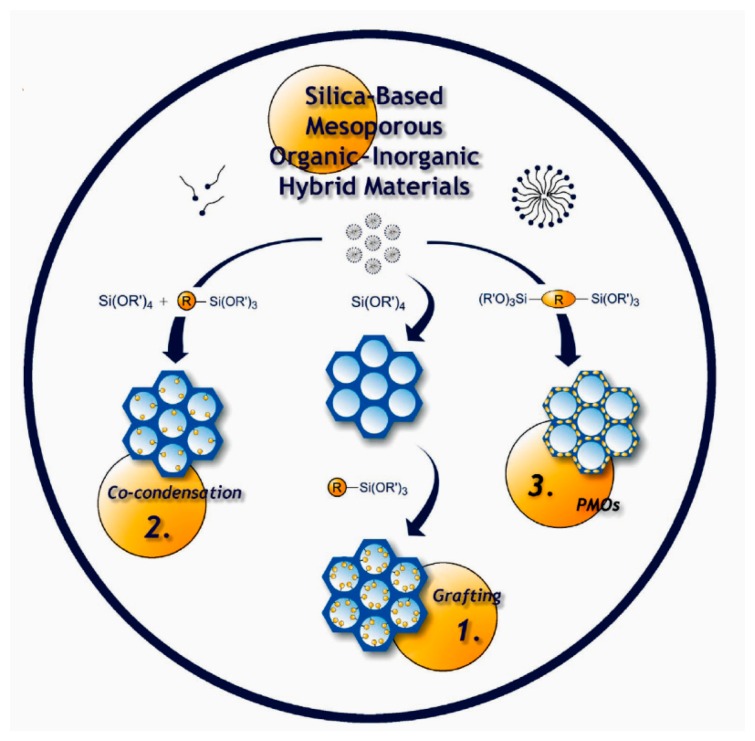
Schematic of three routes for preparing functionalized mesoporous silica. Reprinted with permission from [57].

As is detailed in the literatures and outlined in Figure 7, there are three main routes to introduce organic groups onto the in-wall surface of mesoporous silica: (1) post-grafting; (2) co-condensation and (3) periodic mesoporous organosilicas (PMOs) [57–61]. With any of the above-mentioned methods, mesoporous silica can be functionalized with some desired organic groups to realize satisfied specificity to target molecules. Some typical examples are herein given to illustrate the preparation and applications of the mesoporous silica materials for micro-gravimetric sensing.

### 3.1. HFIP Functionalized SBA-15 Mesoporous Silica for TNT Detection

**Figure 8.**
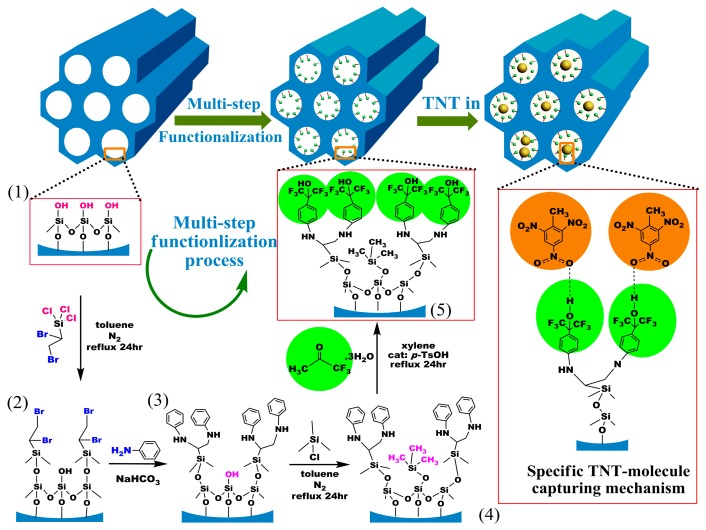
Modification scheme of the HFIP sensing groups onto the inwall of SBA-15 mesoporous-silica and the specific capturing mechanism of the functionalized nano-material to TNT molecules. Reprinted with permission from [34].

It has been confirmed that benzene-ring linked hexafluoroisopropanol (HFIP) possesses the specific hydrogen-bond to nitroaromatic compounds such as trinitrotoluene (TNT) explosive [62–66]. In order to graft HFIP groups onto SBA-15 mesoporous silica, a layer-by-layer in-wall grafting process is designed and shown in Figure 8.

**Figure 9.**
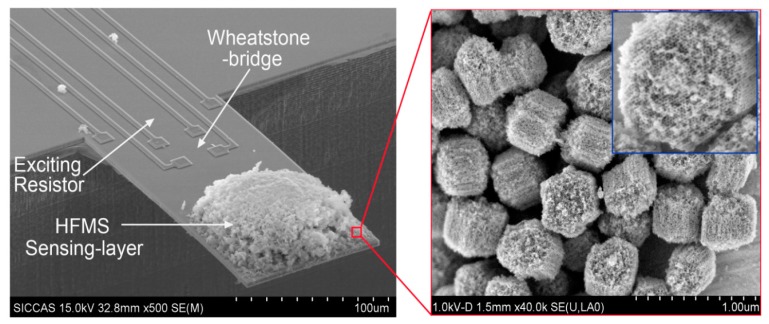
SEM image of the integrated silicon resonant micro-cantilever that is loaded with the HFIP-functionalized mesoporous silica sensing layer. The inset clearly shows the nanoporous structure of the sensing material. Reprinted with permission from [34].

**Figure 10.**
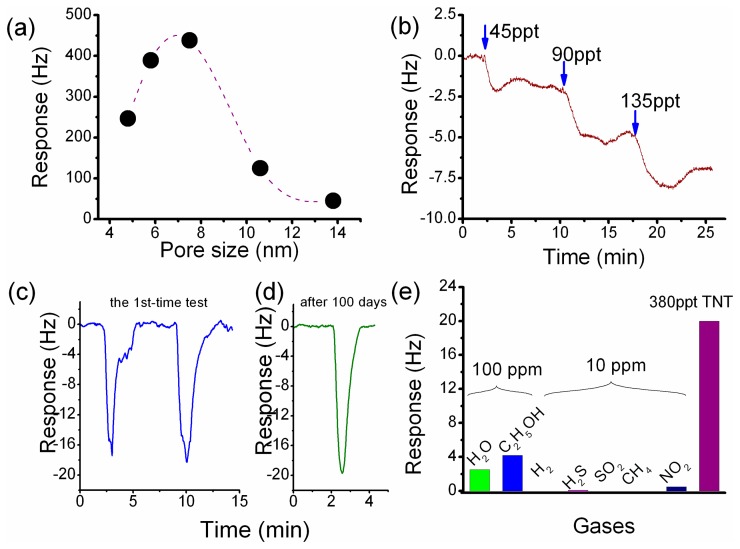
(**a**): Tested effect of pore-size on TNT detection sensitivity; (**b**): Typical sensing response of the functionalized HFMS sensor versus various TNT concentration of 45, 90 and 135 ppt; (**c**): Short-term repeatable and rapidly reversible sensing response to 380 ppt TNT; (**d**): After storing of 100 days, next-time detection with the same sensor shows satisfactory stability in sensitivity; (**e**): Response of the TNT sensor to various kinds of 10–100 ppm interfering gases compared with that to 380 ppt TNT. Reprinted with permission from [34].

After the functionalized sensing material of HFIP-grafted SBA-15 (denoted as HFMS) is prepared, the as-prepared HFMS is dispersed in deionized water under ultrasonic, and then loaded onto the top-surface of our developed resonant micro-cantilever by using an Eppendorf made micro-manipulator. Figure 9 shows the HFMS-loaded cantilever that has been with the resonance-exciting heater and the signal-readout piezoresistors integrated. According to our characterization results shown in Figure 10a, the optimal pore size of the sensing nano-material is 7–8 nm. As is shown in the sensing experiment results of Figure 10b, about a 2.6 Hz frequency drop is obtained when the sensor is exposed to 45 ppt TNT vapor. Compared to the 0.4 Hz noise-floor, the tested sensing signal is about six times in amplitude. When the concentration of TNT vapor increases to 90 ppt and 135 ppt, the corresponding outputs increase linearly. As the reproducible response of 18 Hz to 380 ppt TNT demonstrated in Figure 10c shows, the repeatability of the sensor is quite good. According to the research, the sensor shows fast response/recovery performance, e.g., the responding time to TNT is only about 1 min and the signal recovering time is about 2 min. Due to the covalent-bond graft, the long-term stability of the sensor secures reliable application for more than 100 days (see Figure 10d). The selectivity of the HFIP grafted SBA-15 to several kinds of interfering gases is shown in Figure 10e that indicates satisfactory results. According to the measured data, the TNT sensor exhibits lower than 5 Hz response to all the interfering gases of water (H_2_O), ethanol (C_2_H_5_OH), hydrogen (H_2_), hydrogen sulfide (H_2_S), methane (CH_4_), nitrogen dioxide (NO_2_) and sulfur dioxide (SO_2_). It is worth notifying that the concentration of the interfering gases is herein 10–100 ppm while the TNT concentration is only 380 ppt.

### 3.2. −COOH Functionalized Mesoporous Silica for Ammonia/Amine Detection

Since there is a strong interaction between -COOH group and amine molecule *via* base-to-acid reaction, -COOH group functionalized mesoporous silica (denoted as CMS) is an ideal ammonia/amine sensing material for resonant micro-cantilever detection. Herein, the interference from other natural gases such as CO_2_ can be ignored, as there are few kinds of basic gas (except for ammonia/amines) existing in environment [34,55]. Using CES (carbomethoxysilanetriol, sodium salt, 25 wt% in water) as the key precursor, the CMS sensing material can be prepared with post-grafting method. Using CMS as micro-gravimetric sensing nano-material for NH_3_ detection, five step-increased vapor concentrations (from 0–1 ppm, with 200 ppb increment for each step) are sequentially introduced to the sensor. The frequency signal of the sensor linearly drops by about 7 Hz for every step. After the sensor is switched from the 1 ppm NH_3_ to fresh air, the recovering time of the sensor is shorter than 5 min. Since trimethylamine (TMA) has a higher molecule-weight and larger gas-phase basicity than NH_3_, the same cantilever sensor outputs much higher response to TMA than that to NH_3_. The sensitivity of the sensor approaches about 13 Hz/100 ppb to TMA. According to the research, the limit of detection (LOD) of the sensor to TMA is as fine as about several *tens of ppb*.

### 3.3. −NH_2_ Functionalized Mesoporous Silica Film for CO_2_Detection

**Figure 11.**
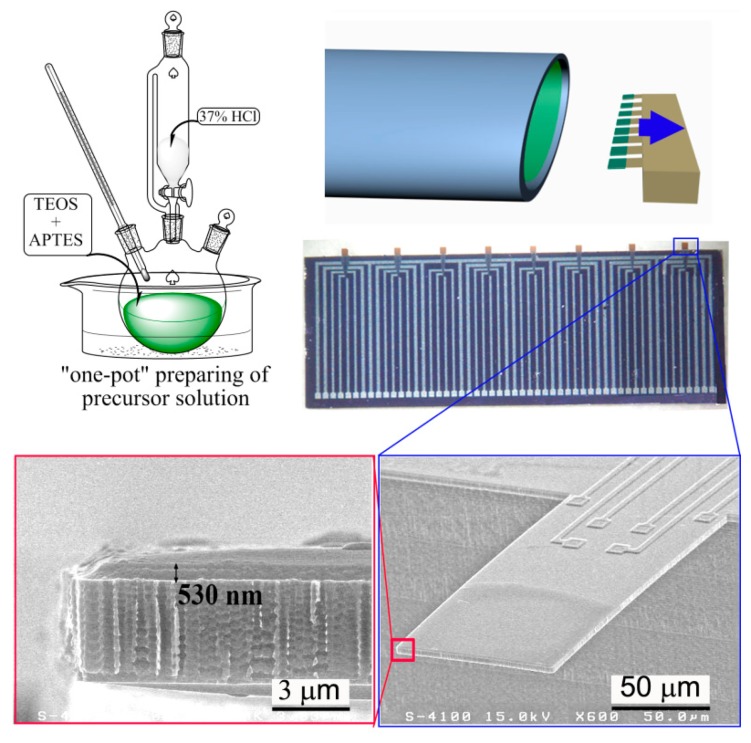
Schematic of −NH_2_ functionalized mesoporous thin film (MTF) batch constructed on the cantilevers and the SEM images of the as-functionalized cantilever sensor. Reprinted with permission from [35].

Similar to the used −COOH functionalized mesoporous silica as sensing material to trace ammonia/amine vapor, −NH_2_ functionalized mesoporous silica can be also used to detect acidic gas like CO_2_ [35,67–69]. Inspired by the EISA (evaporation-induced self-assembly) route for preparing mesoporous thin-film (MTF) on silicon wafer [70–73], our group develops a batch producible nano-on-micro constructing method to directly self-assemble functionalized mesoporous thin-film on the sensing region of the integrated micro-cantilever. Figure 11 schematically shows the procedure of the nano-on-micro constructing technique. Firstly, the precursor is prepared with a so-called “one-pot” method. The as-prepared sol solution is then injected into a specially prepared glass tube. Thereafter, a row of the fabricated cantilevers are with their free-ends simultaneously immersed into the solution for about 5 s and carefully withdrawn using a commercial micro-manipulator (PatchMan NP2, Eppendorf). The cantilevers are always held horizontally in order to ensure that the shear induced alignment of the grown mesoporous nano-channels is inclined to the cantilever surface. The low concentration of the sol-gel precursor leads to the formation of a sufficiently thin film through dip-coating (with a speed of about 10 μm/s). After the films self-assembled on the batch fabricated cantilevers are carefully dried in air for one day, the whole batch of the cantilevers is rinsed several times with acidic solution to remove the CTAB template. Then, the cantilevers surface coated with the −NH_2_ functionalized MTF are treated with a diluted ammonia solution to active the in-wall functionalized −NH_2_ groups. After being rinsed with deionized water several times and blow dried, the functionalized sensors are ready for detection of CO_2_*via* acid-to-base specific reaction.

**Figure 12.**
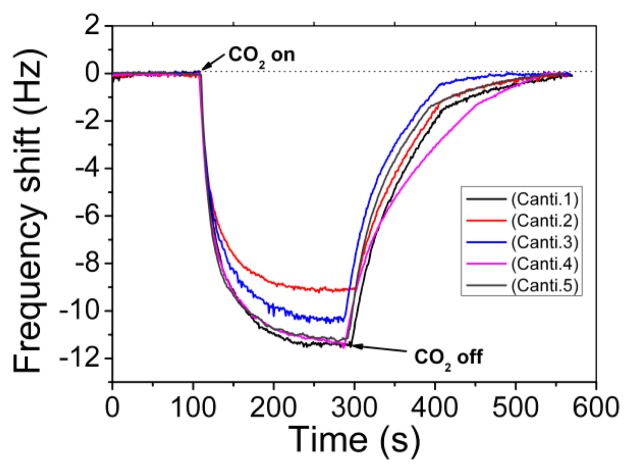
Uniform responses of the five cantilever sensors made in one batch to CO_2_ of 1000 ppm concentration. Reprinted with permission from [35].

Five cantilever sensors are randomly taken from the same batch (denoted as canti. 1–5) as examples, and the consistency of the sensing performance to CO_2_ is well confirmed by the sensing experiment results shown in Figure 12. Before micro-gravimetric detection, the original resonant frequency values of the five cantilever sensors are recorded as 96.285 kHz, 96.291 kHz, 96.299 kHz, 96.303 kHz and 96.310 kHz, respectively. After sequential sensing to CO_2_, the corresponding frequency-shift signals are 9.1 Hz, 10.3 Hz, 11.1 Hz, 11.2 Hz and 11.3 Hz, respectively. The maximum relative error in sensitivity is less than 15%. The satisfactory uniformity verifies that the developed “one-pot” batch MTF-functionalization method can be used in volume production of the nano/micro combined high-performance gas sensors. According to the study, the LOD of the sensor is better than 300 ppm.

**Figure 13.**
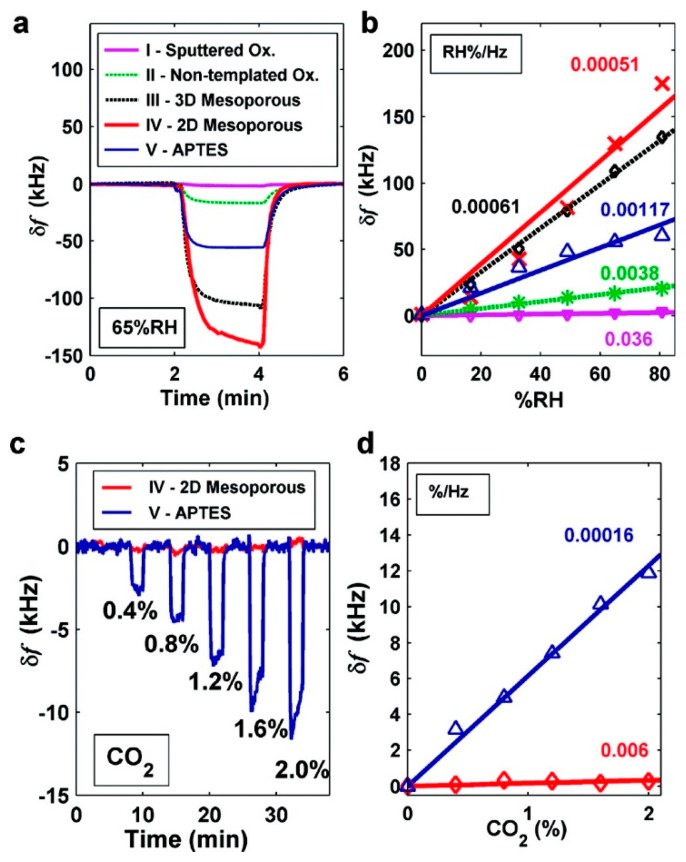
(**a**) Transient frequency shift values of the batch fabricated sensors in response to 65% relative humidity (RH) in N_2_; (**b**) Summary of the maximum frequency-shift sensing signal observed at different RH values; (**c**) Transient frequency shift values of two sensors, number IV and V in (a), in response to various concentrations of CO_2_ in N_2_; (**d**) Plot of the maximum frequency-shift signals observed at different CO_2_ concentrations. Reprinted with permission from [74].

Similar work has also been successfully implemented by Lee and the co-researchers [74,75]. In their work, they also use the EISA method to self-assemble mesoporous silica thin-film onto capacitive ultrasonic transducer (CMUT), which is another kind of resonant sensors fabricated by using micromachined technology. In order to detect humidity change, thermally stable continuous SBA-15 mesoporous silica film are directly grown on the top surface of the batch fabricated CMUT. Compared with the CMUT sensors with the sputtered oxide as humidity sensing material, more than one order of magnitude improvement in sensitivity (5.1 × 10^−4^% RH/Hz) is achieved. In their study, -NH_2_ functionalized MTF is also self-assembled on the CMUT to form CO_2_ sensor. With the results shown in Figure 13, the LOD to CO_2_ gas reaches about 182 ppm.

### 3.4. −SH Functionalized Mesoporous Silica for Heavy Metal Ions Detection

**Figure 14.**
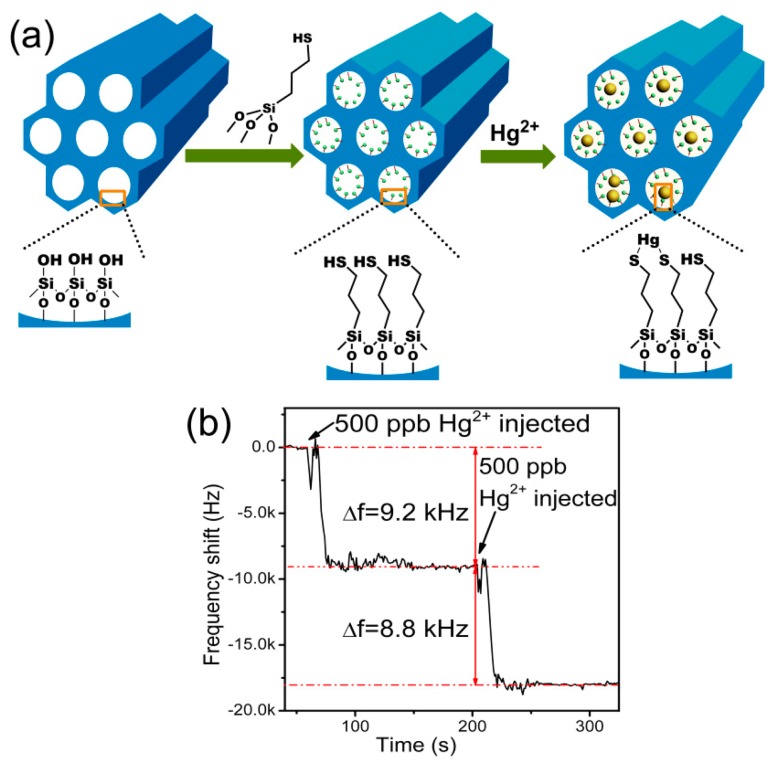
(**a**) Inner channel modification and Hg^2+^ sensing mechanism of functionalized mesoporous silica; (**b**) Real-time detecting data of the sensing-material loaded cantilever for 500 ppb and 1000 ppb aqueous solutions. The Hg^2+^ ion is prepared by injecting Hg (NO_3_)_2_ into aqueous solution. Reprinted with permission from [49].

It is well known that −SH groups can specifically capture Hg^2+^ ions [76–78]. Based on this principle, we use −SH functionalized mesoporous silica as mass-type sensing material to detect Hg^2+^ in aqueous solution [49]. As depicted in Figure 14a, −SH groups are functionalized onto the channel in-wall surface of the mesoporous silica. Then, the sensing material is loaded onto the gold-pad surface of the in-plane resonance-mode cantilever, which was already discussed in Section 2 of this paper. Like the online recorded frequency-signal shown in Figure 14b, the specifically adsorbed mass of Hg^2+^ induces a frequency decrease of about 9 kHz (in detail, it is 9.2 kHz and 8.8 kHz for the sensor exposed to Hg^2+^ of 500 ppb and 1000 ppb concentrations, respectively. Compared to the noise floor from the recorded baseline signal, noise-limited resolution of the ion sensor is estimated to be finer than 100 ppb. In fact, the micro-gravimetric sensing material of the −SH functionalized mesoporous silica can also be used for other gravimetric type sensing platform like QCM [79].

### 3.5. Streptavidin Functionalized Mesoporous Silica for Double-Strand DNA Identification

**Figure 15.**
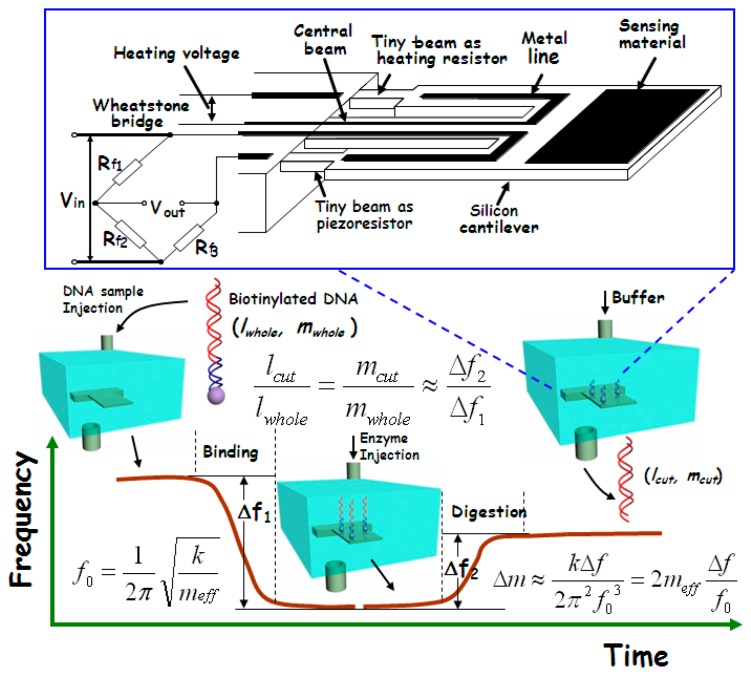
Schematic and procedure for direct identification of double-strand DNA. The in-plane mode resonant cantilever is embedded in a microfluidic chip for real-time DNA detection. The biotin-labeled DNA sample is specifically bound to the cantilever surface via specific binding with pre-immobilized streptavidin. Then, the adsorbed double-strand DNA can be site-specific digested by restriction-enzyme and the corresponding segment of the double-strand chain is cut-off. Two frequency-shift signals can be readout for the DNA binding induced mass increase and the subsequent mass loss due to enzyme digestion, respectively. The two signals can be used for calculating the specific restriction site, thereby directly identifying the double-strand DNA. Reprinted with permission from [81].

In gene-level detection, double-strand DNA is more stable than single-strand DNA and, thus, is more suitable for quick and accurate analysis. However, the conventional double-strand DNA detection methods like electrophoresis are generally time-consuming and expensive [80]. Since the molecular weight of the double-strand DNA fragments (after gel electrophoresis) always needs to be compared with the molecular weight marker, our group recently developed a new micro-gravimetric method to recognize double-strand DNA [81]. According to the restriction enzyme effect, there are one or more specific sites, along the chain of a certain double-strand DNA, for digestion of corresponding restriction enzyme. After digestion by an enzyme, the double-strand will be cut into two segments with a certain ratio in length. Since this ratio of length is proportional to the ratio of mass between the two segments, the enzyme digestion site can be identified by measuring the relative mass change after the site-specific enzyme cutting-off. This new idea has been realized with our micro-gravimetric sensing platform, where the mesoporous silica with huge surface area has been used to improve the binding of double-strand DNA sample on the resonant sensor. The sensing scheme is outlined in Figure 15.

**Figure 16.**
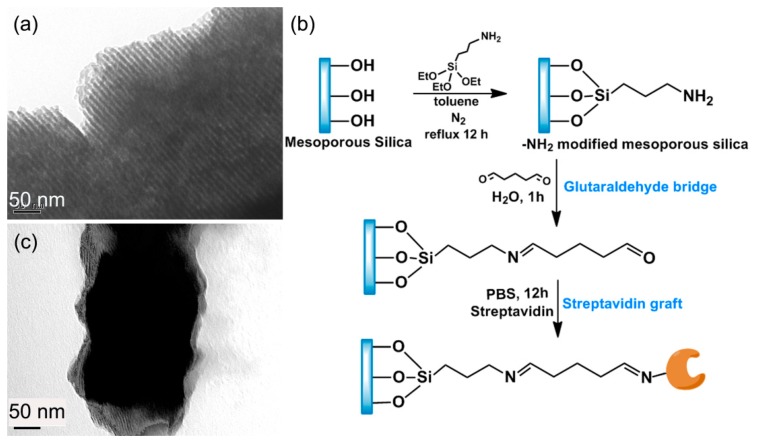
(**a**) TEM image showing ordered nano-structure of the mesoporous-silica raw material; (**b**) Schematic route of functionalizing streptavidin onto the mesoporous-silica; (**c**) TEM image of the streptavidin-functionalized mesoporous silica. Reprinted with permission from [81].

Herein, the sample immobilization of the double-strand DNA is completed by using the huge surface area mesoporous-silica as media material. The pore diameter of the used mesoporous-silica raw material is about 10 nm, as depicted in the TEM image of Figure 16a. Pre-functionalization of streptavidin onto the mesoporous silica is implemented with the following steps. 3-aminopropyl-triethoxysilane (APTES) is firstly introduced to react with the surface silanol-group to obtain −NH_2_ functionalized mesoporous-silica. Then, glutaraldehyde is reacted with the −NH_2_ group to form an aldehyde tail. After that, streptavidin is grafted on top of the aldehyde group. The route of the streptavidin functionalization onto the mesoporous-silica surface is shown in Figure 16b. The morphology of the streptavidin-functionalized mesoporous-silica is illustrated in the TEM image of Figure 16c.

**Figure 17.**
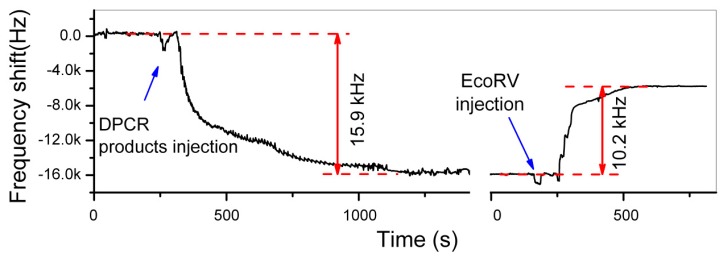
Results for experimental identification of double-strand DNA of *E. coli O157:H7* and three control experiments. After the PCR products of *E. coli O157:H7* (stx2F/Bio-stx2R) is injected into the detection chamber, the cantilever resonance frequency continually decreases that indicates the mass addition due to the binding of the biotinylated DNA onto the mesoporous-silica surface. After the enzyme is injected, the frequency reversely increases to reflect the enzyme digestion induced mass loss in real-time. Reprinted with permission from [81].

The double-strand DNA direct detection method to the pathogen of *E. coli O157:H7* has been experimentally verified by detection of the 3776-bp chain that contains stx2 gene of *E. coli O157:H7*. The site-specific digestion with the enzyme of *EcoRV* (at 2635-bp) is experimentally implemented, resulting in accurate frequency signal for DNA identification. Figure 17 shows the real-time recorded frequency response of the resonant cantilever during the whole processes of the 3776-bp double-strand DNA bound on the cantilever and the enzyme specific-cutting at the restriction site of 2635-bp of the chain. After the PCR (polymerase chain reaction) product of biotinylated DNA (with 1 ng/μL concentration) is injected into the detection chamber, the frequency of the cantilever continually decreases along with the process of the DNA double-strands bound onto the functionalized mesoporous-silica that has been pre-loaded on the cantilever. The frequency reaches to a stable state in 10 min that indicates completion of DNA sample immobilization. The frequency-shift value of *Δf_1_* = 15.9 kHz was recorded, which indicated 1.8 ng DNA bound onto the cantilever. After the sensor is balanced by injecting the buffer of *EcoRV* enzyme from the inlet of the detection chamber, the *EcoRV* solution is introduced into the chamber. During the site-specific digestion process, the frequency of the micro-cantilever rapidly increases by *Δf_2_* = 10.2 kHz. Thus, the experimentally obtained ratio between the two frequency-shift values is measured as *Δf_2_*/*Δf_1_* = 64.2%. Due to that the adsorbed mass is much smaller than the effective mass of the resonant cantilever itself, the ratio of *Δf_2_*/*Δf_1_* should be equal to the ratio between the mass of the bound DNA and the cut-off mass by enzyme. Since the mass per DNA base-pair (bp) is a constant, *Δf_2_*/*Δf_1_* should be also equal to the ratio between the strand-length changes during the DNA immobilization and the enzyme cutting, *i.e.*, *l_cut_*/*l_whole_* = 64.2%. According to the known DNA cutting site of 2635-bp from the original 3776-bp chain, the theoretical mass ratio between the removed DNA segment and the whole DNA chain should be *l_cut_*/*l_whole_* = 2635/3776 = 69.8%. With a small part of uncompleted enzyme digestion taken into account, the measured ratio of 64.2% can be considered being consistent with the theoretical ratio of 69.8%. Hence, this quantitative method for identifying the double-strand DNA is experimentally verified. When the purified DNA sample of identical concentration is used in the detection experiment, it still generated similar detecting results. Thus, there is no need to purify the PCR products when this detection technique is employed.

## 4. Nanoporous Zeolites

As an important kind of nanoporous material, zeolites have been intensively researched in design, synthesis and application [8,82–84]. Featuring molecular scale nanopores (about 3–15 Å) and ultra-high specific surface area, zeolites have been utilized as sensing materials for many years [85]. Zhou and her co-workers successfully coated MFI-type zeolites onto piezoelectric micro-cantilevers to form resonant gas sensors [86]. The zeolite-modified micro-cantilever sensor can be used to detect 100 ppm Freon gas. In another work, Urbiztondo *et al.* reported that Co-BEA (Co^2+^ exchanged Beta) type zeolite film is directly deposited onto a resonant micro-cantilever [87]. Using the Co-BEA type zeolite modified micro-cantilever sensor, 1 ppm nitrotoluene has been detected with a high selectivity.

Among various types of zeolite nano-materials, we need to pay attention to the nanoporous material of zeolitic imidazolate frameworks (ZIFs), since the material is a very popular topic in the research fields of gas molecule purification, capturing and storage [88–93]. In addition to the huge surface area and chemical/thermal stability, the ZIFs feature high gas affinity and high uptake capacity to CO_2_ molecules [94]. Especially for industrial applications, the ZIF material shows technical advantage in highly selective CO_2_ adsorption [95].

**Figure 18.**
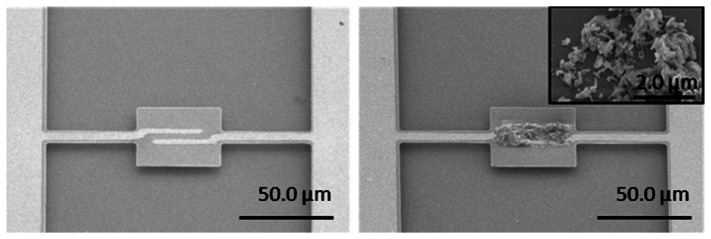
SEM images of the fabricated resonator (**left side**: without the ZIFs; **right side**: with the ZIFs. Reprinted with permission from [97].

**Figure 19.**
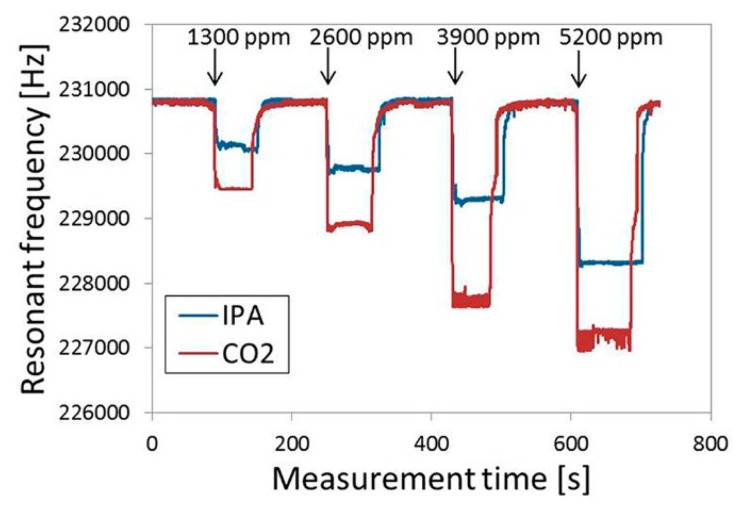
Real-time gas sensing response of the ZIF-loaded resonator to IPA and CO_2_. Reprinted with permission from [97].

Hwang and the co-workers have reported research on ZIF-coupled micro-resonators for highly sensitive and selective gas detection [96,97]. Dielectrophores is (DEP) is used to self-assemble ZIF-69 nanoparticles onto the as-fabricated resonator. The fabricated sensor and the sensing material are shown in the SEM images of Figure 18. In the study, the ZIF-coupled resonator chemical sensors are successfully used to detect IPA (isopropyl alcohol) and CO_2_, with the results shown in Figure 19. According to the report, compared with the bare silicon resonator, the amount of CO_2_ molecule number adsorbed onto the ZIF-loaded resonator is enhanced more than 70 times. The results verified that the ZIFs can significantly increase the surface area of the sensor for gas detection.

## 5. Nanoporous Hierarchical Sheets of Graphene-Oxide/Gold-Nanoparticles

With ultra-high intrinsic specific-surface-area (e.g., the theoretical value approaches about 2630 m^2^/g [98–101]), graphene sheets show great promise in micro-gravimetric sensing applications. Moreover, for detection of environmental gases, the hydrophobic nature of graphene [102–105] can effectively depress the interfering noise from moisture adsorption. However, due to the strong π-π stacking and van der Waals force [106], the mono-layered graphene sheets tend to aggregate. After dried from solution, the aggregation effect often results in the formation of compressed graphene paper. The aggregation of graphene sheets dramatically decreases the effective specific surface-area of the nano-material from 2630 m^2^/g to lower than 10 m^2^/g. After aggregation, the interlayer gap distance of the graphene sheets will reduce to 0.34–0.44 nm [107,108], which is inaccessible for many chemical molecules (e.g., the molecule diameter of nitrogen is larger than 0.364 nm) to be transported into the inner space of the graphene sheets. In such case, the chemical molecules can only be adsorbed on the outer surface of the graphene paper. Hence, for micro-gravimetric sensing applications, it is essential to keep the intrinsic high surface area of pristine graphene.

**Figure 20.**
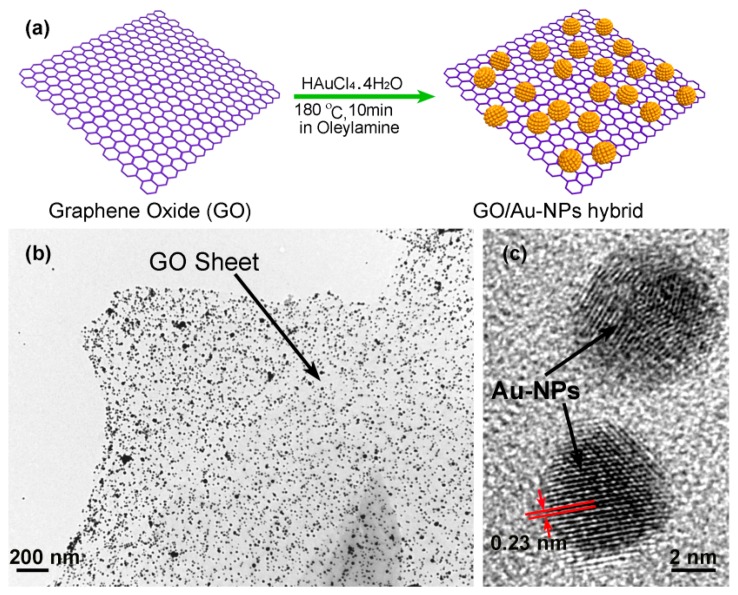
(**a**) Schematic process of Au-NPs grown on a GO sheet; (**b**) TEM image of the GO/Au-NPs hybrid; (**c**) High-resolution TEM (HR-TEM) image showing two Au-NPs on the GO sheet. Reprinted with permission from [109].

In our group, we use an *in-situ* method to self-assemble gold nanoparticles (Au-NPs) onto the graphene nanosheets, thereby constructing nanoporous-layered three-dimensional (3D) hierarchical graphene foam after solvent removal [109]. Figure 20a shows the preparing process of the GO/AuNPs sample, where oleylamine plays the key reactant and solvent. It is known that there exists a strong interaction between the −NH_2_ group in oleylamine and the oxygen-containing group (like −COOH, −OH or epoxy group) in GO sheet. Besides, the similarity between olefin group in oleylamine and benzene-ring in GO sheet enhances inter-miscibility of GO in oleylamine. Under stirring, the compressive GO material with accurate mass is easily dispersed in oleylamine to form a stable and high-concentration GO dispersion. Thereafter, the gold precursor of HAuCl_4_·4H_2_O is added into the dispersion and, then, AuCl_4_^−^ ions are adsorbed onto the GO surface via coordination effect between the oxygen-containing group in GO and the AuCl_4_^−^ ion. With the help of the olefin group in oleylamine, the gold ions are reduced to form Au-NPs at high temperature. The TEM characterization results shown in Figure 20b indicate that the well dispersed Au-NPs are *in-situ* grown onto the GO sheets. The high resolution TEM (HR-TEM) image in Figure 20c clearly shows two Au-NPs (with about 5 nm in diameter), where the crystal lattice fringes of 0.23 nm match well with the (111) plane of gold. In addition, the density of the Au-NPs decorated on the surface of the GO sheet can be tuned conveniently by adjusting the weight ratio between GO and HAuCl_4_·4H_2_O in the range of 0.15–1.3. In our experiment, the weight ratio of Au-NPs in the hybrid can be up to 60 wt%. In the nanoporous graphene material, the Au-NPs are used as building blocks to prevent graphene sheets from aggregation. Using this method, the highly effective specific surface-area can be kept. After that, functional organic groups can be grafted either on the activate sites of the graphene or on the surface of Au-NPs for chemical sensing.

### 5.1. HFIP (Hexafluoroisopropanol) Functionalized GO/Au-NPs Nanoporous Sheets

**Figure 21.**
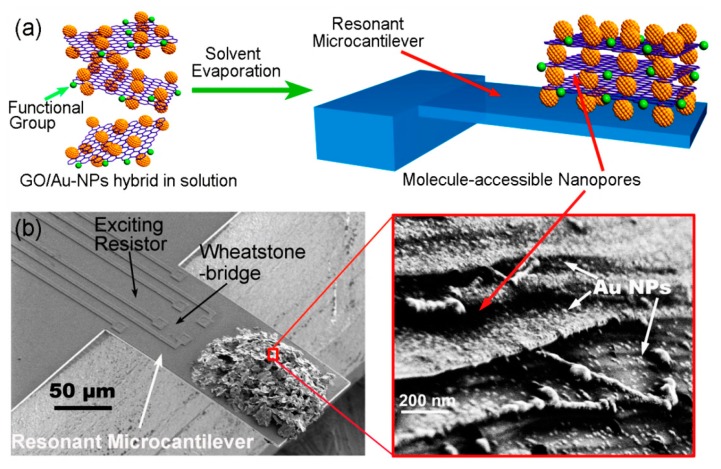
(**a**) Schematic showing the formation of the GO/Au-NPs nanoporous sensing material; (**b**) SEM image of the micro-cantilever with the porous-layered GO hybrid *in-situ* constructed. The multiple porous-layered stacks can be viewed in the inset close-up with SEM. Reprinted with permission from [109].

With the GO/Au-NPs hierarchical foam as raw material, carbonyl diimidazole (CDI) is selected as coupling reagent to graft HFIP (hexafluoroisopropanol) sensing groups onto the oxygen-containing active sites of GO sheets. Then, the HFIP functionalized GO/Au-NPs material is loaded onto the resonant micro-cantilever, by using the same method described in Section 3.1. Due to the pillar supporting effect of the Au-NPs, the stacked GO sheets are then able to take on a multi-layered nanoporous structure (see Figure 21). Aided by a lab-made PLL (phase-locked loop) close-looped interface circuit, the resonant micro-cantilever can be used as a mass-type chemical sensor to effectively adsorb and detect TNT vapor in real-time.

**Figure 22.**
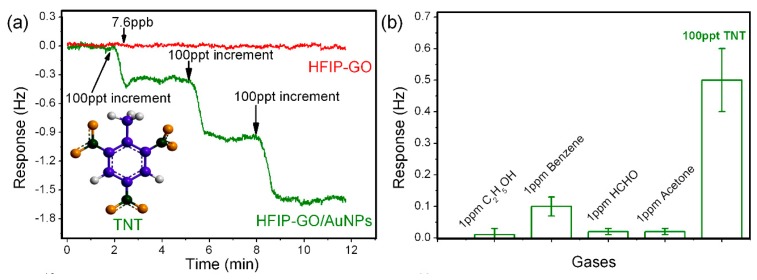
(**a**) Sensing response of the sensor to TNT at concentrations of 100, 200 and 300 ppt; (**b**) Responses of the TNT sensor to 1 ppm interfering gas is compared with that to 100 ppt TNT for evaluation of selectivity. Reprinted with permission from [109].

Before TNT detection, the resonant frequency of the micro-cantilever is measured as about 75 kHz in air, with the signal noise-floor being lower than 0.1 Hz. As is shown in Figure 22a, when TNT vapors with the concentrations of 100 ppt, 200 ppt and 300 ppt are sequentially exposed to the sensor, step-by-step frequency shifts are measured, with an average sensing response of about 0.5 Hz to 100 ppt increment of TNT. According to the known 1.53 Hz/pg mass sensitivity of the resonant sensor, the mass of 100 ppt TNT molecules adsorbed on the HFIP-functionalized GO nanoporous sensing material can be worked out as 327 fg. Due to the covalent-bond grafting of the HFIP groups, the long-term stability of the sensor is quite satisfactory. After storage for six months, the repeated TNT detection with the same sensor results in negligible decrease in sensitivity. To investigate selectivity, several common vapors, such as ethanol, acetone, formaldehyde and benzene, are selected as interfering gases for experimental comparison. As is shown in Figure 22b, the HFIP-functionalized GO/Au-NPs nanoporous sensing material shows a good selectivity to TNT of 100 ppt concentration, while all the interfering vapors are with a much higher concentration of 1 ppm. The ultra-high selectivity may be attributed to the highly specific interaction between HFIP group and TNT molecule, which has been proved in the previous literatures [63].

In our control experiment, traditional GO paper (*i.e.*, without the Au-NPs grown on the sheet) is also functionalized with HFIP and loaded on another identically structured micro-cantilever for experiment of TNT adsorbing and sensing. In that case, the cantilever shows no obvious sensing response even when the saturated TNT vapor (with the concentration of about 7.6 ppb) is introduced (also see Figure 22a). Without the nanoporous treatment, the molecule inaccessible compressive structure features a much lower specific surface-area and, thus, only a small number of TNT molecules can be adsorbed at the outer surface that leads to the negligible micro-gravimetric response.

### 5.2. −COOH Functionalized Au-NPs/rGO Sheets

**Figure 23.**
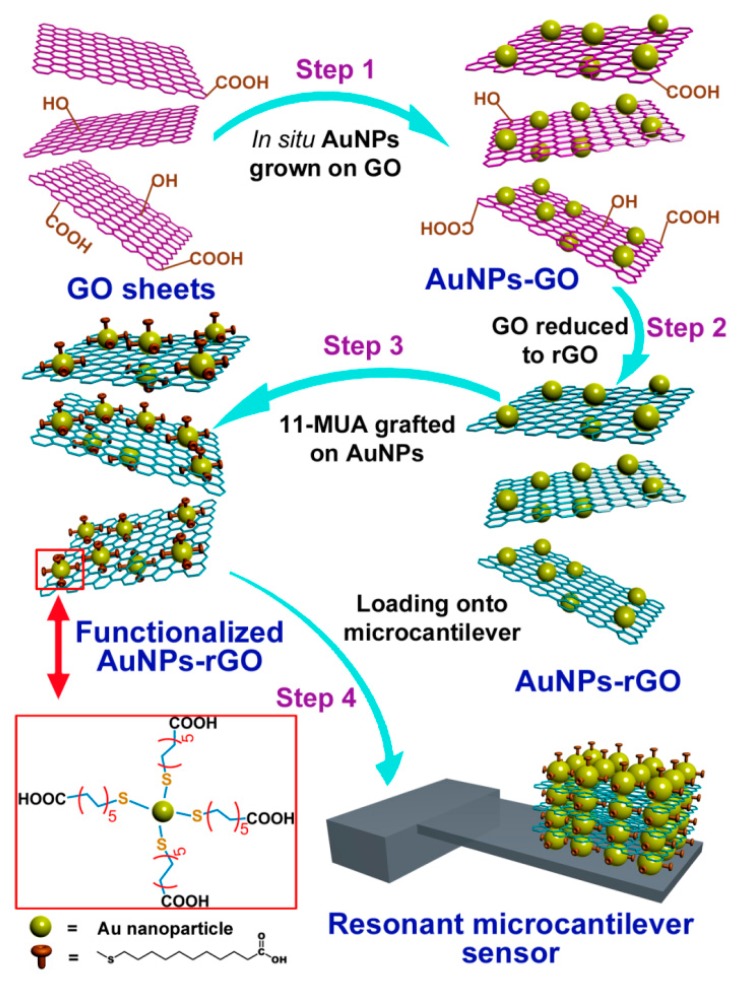
Schematic of the process for preparing functionalized AuNPs-rGO and constructing the nanoporous material on a cantilever. Reprinted with permission from [110].

Instead of grafting organic groups onto the oxygen-containing active sites of GO sheets (mainly at the edges of the GO sheets), surface modification of organic groups onto noble metal (e.g., gold) is the alternative functionalization approach [110–112]. With the alternative method, the functional groups can be grafted onto the metal nanoparticles instead of the GO sheet itself. Thus, in order to prepare sensing materials with hydrophobic characteristic, the hydrophilic GO sheets bearing the oxygen-containing groups should be transformed back to rGO (reduced graphene oxide) that becomes hydrophobic again. To obtain rGO/Au-NPs, ascorbic acid (Vitamin C) is used as reducing reagent to treat the raw material of GO/Au-NPs. Then, −COOH groups can be grafted onto Au-NPs via the well-known self-assembly method that has been detailed in the previous literatures [113]. Briefly, 11-mercaptoundecanoic acid (11-MUA) is firstly dissolved into ethanol to form a stock solution, where a small amount of H_2_O is added as catalyst. Then, AuNP–rGO hybrid, which is pre-activated for 3 min under O_2_ plasma, is immersed into the stock solution for about 8 h. The samples are then sequentially filtrated and rinsed with ethanol and distilled water for several times to remove the chemical residues. After drying under vacuum, −COOH functionalized nanoporous material can be finally obtained. The whole process of the sample preparing and sensor construction is schematically shown in Figure 23.

**Figure 24.**
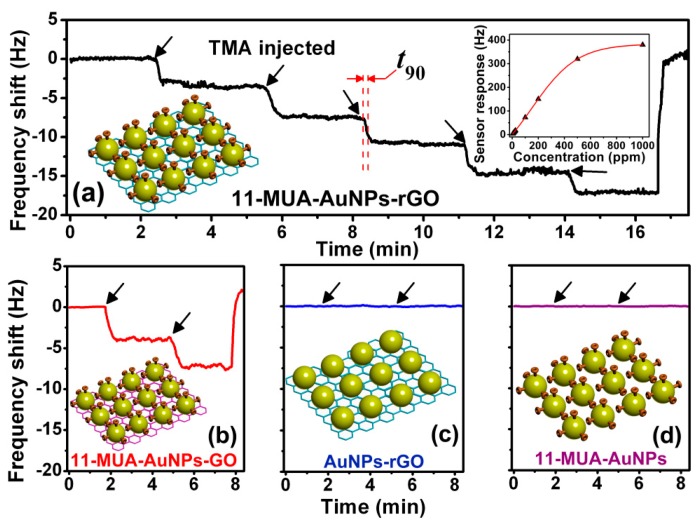
Measured frequency sensing signals to TMA vapors. The cantilever sensors are loaded with different sensing materials: (**a**) functionalized AuNPs-rGO; (**b**) functionalized AuNPs-GO; (**c**) AuNPs-rGO without functionalization; (**d**) functionalized AuNPs. The arrows denote the moments when stepwise-increased TMA vapors (increment is 5 ppm) are sequentially injected into the testing chamber. Reprinted with permission from [110].

To form a micro-gravimetric sensor for amine detection, −COOH functionalized nanoporous sensing material is prepared and *in-situ* stacked at the free end of a resonant cantilever. As is shown in Figure 24a, the sensor outputs serial frequency-shift signals when it exposed to TMA vapor with stepwise increased concentration. The sensing signal of the sensor is about 3.5 Hz for 5 ppm TMA. Additionally, there is no obvious attenuation in sensitivity during the multi-concentration detection. Figure 24b shows that, compared with the sensor loaded with 11-MPA functionalized AuNPs-GO (*i.e.*, the GO is not reduced back to rGO), the TMA detection sensitivity of the current sensor with AuNPs-rGO is almost the same. Due to the nanoporous structure of the sensing material, the response of the sensor is very rapid with the response time (*t_90_*) being shorter than 30 s, where *t_90_* is defined as the time period corresponding to the output signal amplitude reaching 90% of the maximal output value. The recovery of the sensing signal to its original baseline is also less than 30 s after the multi-concentration detection. The AuNPs-rGO without functionalization and the 11-MUA functionalized AuNPs are also used for the TMA sensing experiment. The sensing results show negligible response to 5 ppm TMA (see Figure 24c,d). The results verify that the sensing effect originates from the sensing-group functionalization.

**Figure 25.**
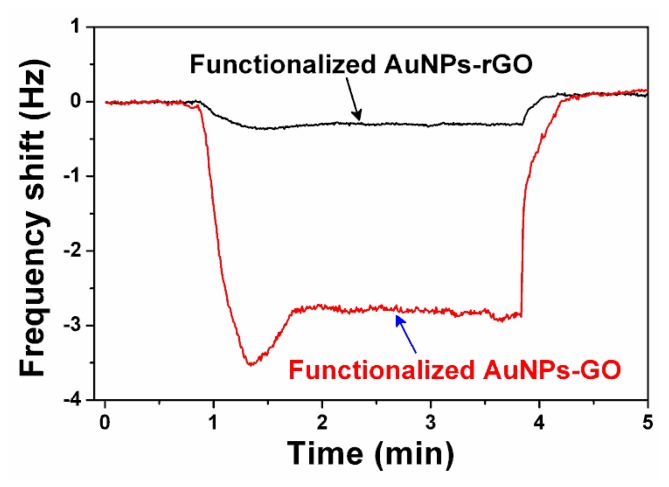
Under the same humidity change (by vaporizing injected water in the detection chamber), the sensor with the functionalized AuNPs-rGO sensing material shows a much lower response compared with the sensor loaded with the functionalized AuNPs-GO. Reprinted with permission from [110].

The hydrophobic AuNPs-rGO material experimentally shows much better depression effect to environmental moisture than AuNPs-GO. Figure 25 shows the experimental comparison between the two materials. Under the same 100 ppm concentration change of H_2_O vapor, the AuNPs-rGO sensor responses 0.4 Hz that is much lower than the 2.8 Hz of the AuNPs-GO sensor. Compared with the AuNPs-GO material (with contact angle of 61.6°), the higher hydrophobic property of the AuNPs-rGO material (with contact angle of 131.5°) indeed contributes to the low-noise performance.

**Figure 26.**
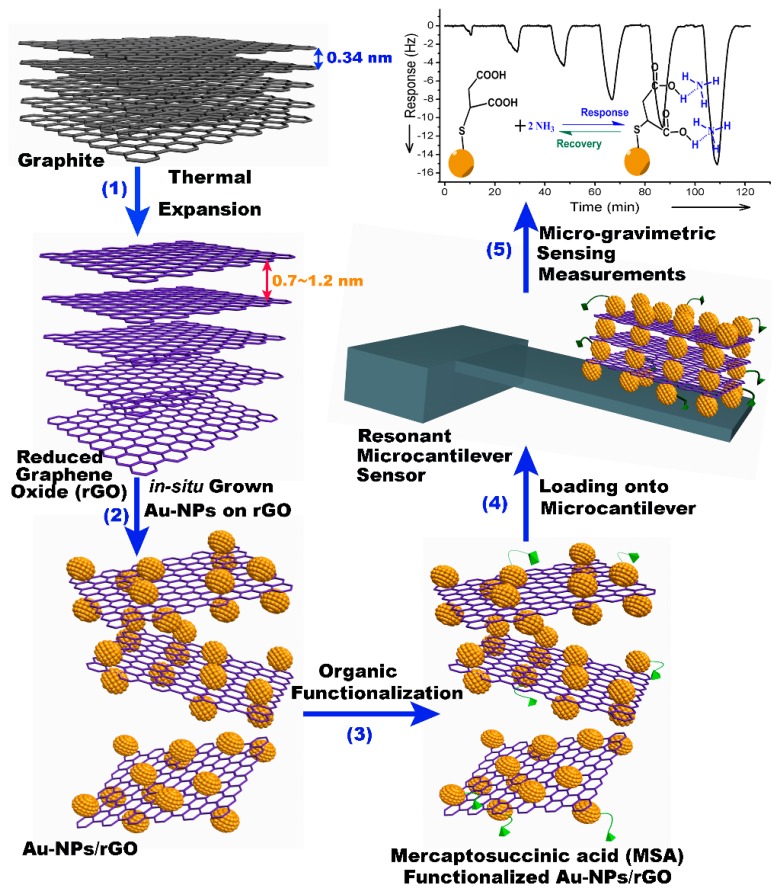
Schematic showing the process for preparing −COOH functionalized Au-NPs/rGO and constructing cantilever NH_3_ sensor. Reprinted with permission from [114].

More recently, we developed a facile thermal expansion route to prepare the hydrophobic rGO material to replace the aforementioned multi-step Vitamin C reduction approach [114]. To obtain the graphite-oxide, we firstly use an acid mixture to oxidize commercial-available graphite powder. Then, the graphite-oxide product is exfoliated by thermal shock on rapid exposure to temperature of 900 °C in inert atmosphere. During this process, the thermal vaporization of the acid molecules intercalated between the graphite oxide sheets leads to dramatic expansion of the graphite oxide to form the required rGO sheets. Under high temperature, the oxygen-containing functional groups (including epoxide, carboxyl, and hydroxyl groups) in graphite oxide can be thermally decomposed to CO_2_ and H_2_O in the inert atmosphere. After that, the Au-NPs could be also *in-situ* self-assembled on the surface of the rGO sheets. Some organic groups like −COOH group can be grafted onto the Au-NPs for sensing functionalization. Figure 26 schematically shows the whole preparing route of the nanoporous sensing-material.

**Figure 27.**
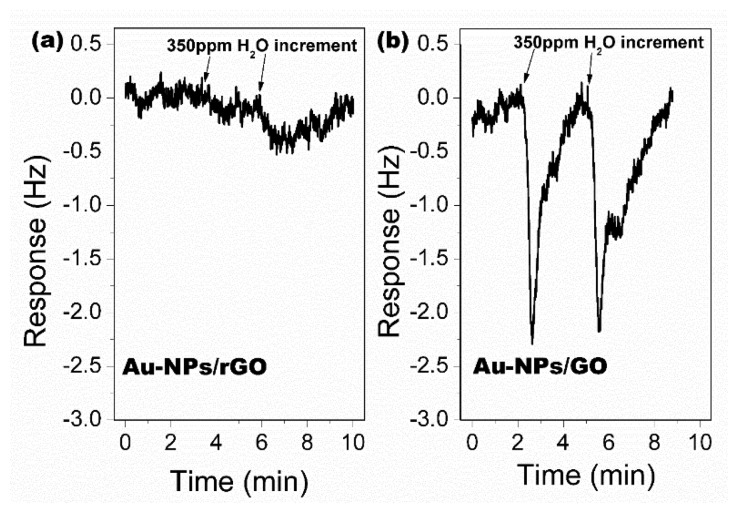
Sensing responses of the sensors loaded with Au-NPs/rGO (**a**) and Au-NPs/GO (**b**) to 350 ppm water. Reprinted with permission from [114].

As described above, the rGO sheets directly obtained from the thermal decomposition method feature higher hydrophobic property than GO, because a large amount of the oxygen-containing hydrophilic groups are decomposed during the pyrolysis process, and the amount of the oxygen-containing groups in rGO is much lower than that in GO. The contact-angle (CA) measurement results indicate that the CA of rGO is about 72.5°, which is in contrast to the CA of GO of 35.6°. The Au-NPs/GO and the Au-NPs/rGO are both used for sensing experiment to water. As is shown in Figure 27, the Au-NPs/GO sensor outputs about a 2 Hz response to 350 ppm water vapor, while the sensor with Au-NPs/rGO generates negligible response to water vapor. The results well confirm that Au-NPs/rGO is advantageous in minimizing the influence from environmental humidity.

## 6. Conclusions and Outlook

In this review, we focus on the advanced nanoporous sensing material for gravimetric sensing platform of resonant micro-cantilevers. Of course, the addressed sensing nano-materials are also suitable for use in other bio/chemical adsorption/sensing tools like QCM. So far, there are several kinds of nanoporous materials that have been successfully used for advanced micro/nano sensors. The applied nanoporous sensing-materials mainly include mesoporous silica, zeolite, nanoporous graphene oxide (GO) and so on. Using functionalized nanoporous sensing material that features a large specific surface area, several kinds of bio/chemical sensors have been constructed for rapid detection of trace-level bio/chemical molecules.

According to the abovementioned achievements and our research experience, the following issues deserve attention in future research and development.

*i*) Gravimetric resonant micro-sensors often suffer from the influence of ambient humidity change, since the sensors are normally operated at room temperature. Thus, design and preparation of hydrophobic nanoporous sensing materials with negligible water response will still be an important topic in the near future.
*ii*) Until now, exploration of functional sensing-groups for highly specific recognition of various gases is lagging behind and, thus, needs to be sped up. Currently, most of the available sensing-groups are sensitive to polar molecules like ammonia or carbon dioxide. How to specifically capture non-polar molecules (e.g., benzene and methane) onto the sensing materials is still a difficult task.
*iii*) New sample preparation technologies should be developed to adapt to various nanoporous sensing-materials. For example, as is reported in a great number of literatures, kinds of MOFs (metal-organic frameworks) nanoporous materials exhibit excellent gas adsorption properties [115]. Thus, MOFs are reasonably expected to serve as micro-gravimetric sensing-materials for chemical detection. Unfortunately, so far it is still difficult to load MOF crystal onto the tiny region of the micro-cantilever sensors.
*iv*) Besides the optimization in sensing materials, the transducer of resonant cantilever needs to be improved for higher sensitivity. With higher resonant modes, the recently developed cantilever sensors have shown adequately high Q factor in atmosphere for gas detection. In liquid, however, relatively much lower Q is still a main obstacle towards finer resolution of bio/chemical analytes in solution. Exploration of new resonating structures with low-damping characteristics is in high demand.

## Figures and Tables

**Table 1.** Categorization of the resonant micro-cantilever [30,31,45].

**Cantilever Material**	**Shapes**	**Exciting Method**	**Read-Out Method**
Si	Square	Electrothermal	Optical leverage
SiN	Triangle	Electromagnetic	Capacitive
SiO_2_	U-shape	Piezoelectric	Piezoelectric
SU-8		Electrostatic	Piezoresistive
Graphene		Laser	Hard contact
Polystyrene			Tunneling

## Acronyms

AC: alternating current
CMUT: capacitive micromachined ultrasonic transducer
deep-RIE: deep reactive ion etching
DEP: dielectrophoresis
EISA: evaporation-induced self-assembly
AuNPs: gold nanoparticles
GO: graphene oxide
IPA: isopropyl alcohol
LOD: limit of detection
MTF: mesoporous thin-film
MOFs: metal-organic frameworks
PECVD: plasma enhanced chemical vapor deposition
Q-factor: quality factor
PLL: phase-locked loop
ppt: parts per trillion
ppm: parts per million
ppb: parts per billion
QCM: quartz crystal microbalance
SAM: self-assembled monolayer
SEM: scanning electron microscopy
SOI: silicon on insulator
TEM: transmission electron microscopy
TMA: trimethylamine
TNT: trinitrotoluene
ZIFs: zeolitic imidazolate frameworks
